# Tremor in Spinocerebellar Ataxia: A Scoping Review

**DOI:** 10.5334/tohm.911

**Published:** 2024-06-20

**Authors:** Adreesh Mukherjee, Sanjay Pandey

**Affiliations:** 1 Department of Neurology and Stroke Medicine, Amrita Hospital, Mata Amritanandamayi Marg Sector 88, Faridabad, Delhi National Capital Region, India

**Keywords:** Ataxia, Cerebellum, Tremor

## Abstract

**Background:**

Spinocerebellar ataxia (SCA) denotes an expanding list of autosomal dominant cerebellar ataxias. Although tremor is an important aspect of the clinical spectrum of the SCAs, its prevalence, phenomenology, and pathophysiology are unknown.

**Objectives:**

This review aims to describe the various types of tremors seen in the different SCAs, with a discussion on the pathophysiology of the tremors, and the possible treatment modalities.

**Methods:**

The authors conducted a literature search on PubMed using search terms including tremor and the various SCAs. Relevant articles were included in the review after excluding duplicate publications.

**Results:**

While action (postural and intention) tremors are most frequently associated with SCA, rest and other rare tremors have also been documented. The prevalence and types of tremors vary among the different SCAs. SCA12, common in certain ethnic populations, presents a unique situation, where the tremor is typically the principal manifestation. Clinical manifestations of SCAs may be confused with essential tremor or Parkinson’s disease. The pathophysiology of tremors in SCAs predominantly involves the cerebellum and its networks, especially the cerebello-thalamo-cortical circuit. Additionally, connections with the basal ganglia, and striatal dopaminergic dysfunction may have a role. Medical management of tremor is usually guided by the phenomenology and associated clinical features. Deep brain stimulation surgery may be helpful in treatment-resistant tremors.

**Conclusions:**

Tremor is an elemental component of SCAs, with diverse phenomenology, and emphasizes the role of the cerebellum in tremor. Further studies will be useful to delineate the clinical, pathophysiological, and therapeutic aspects of tremor in SCAs.

## Introduction

Autosomal dominant spinocerebellar ataxia (SCA) consists of an ever-expanding list of diseases with the common theme of cerebellar ataxia with a genetic etiology. From the initial descriptions of SCA, it was evident that symptoms and signs beyond ataxia comprised a substantial part of the clinical repertoire. An action tremor in the presence of evident ataxic features is linked to the cerebellar pathology and accompanies several SCAs. The clinical scenario becomes intriguing when the patient presents with a postural tremor in the absence of significant ataxia, or with a predominant rest tremor. Notwithstanding the initial presentation, the evolution of symptoms often leads to a troubling tremor in SCA patients, which impairs their functional status. Hence, tremor forms an essential component of SCAs. This also presents the opportunity to understand the role of the cerebellum and its connections in the pathophysiology of tremor. However, a comprehensive discussion on the prevalence, phenomenology, and pathophysiology of tremor in SCAs is lacking. Current updates on SCAs pertaining to the clinical and pathophysiological aspects do not elaborate on tremor and its correlates [1,2,3,4]. Hence, the present review aims to describe the various types of tremors seen in the different SCAs, with a discussion on the pathophysiology of the tremors, and the possible treatment modalities.

## Methods

**Figure 1:**
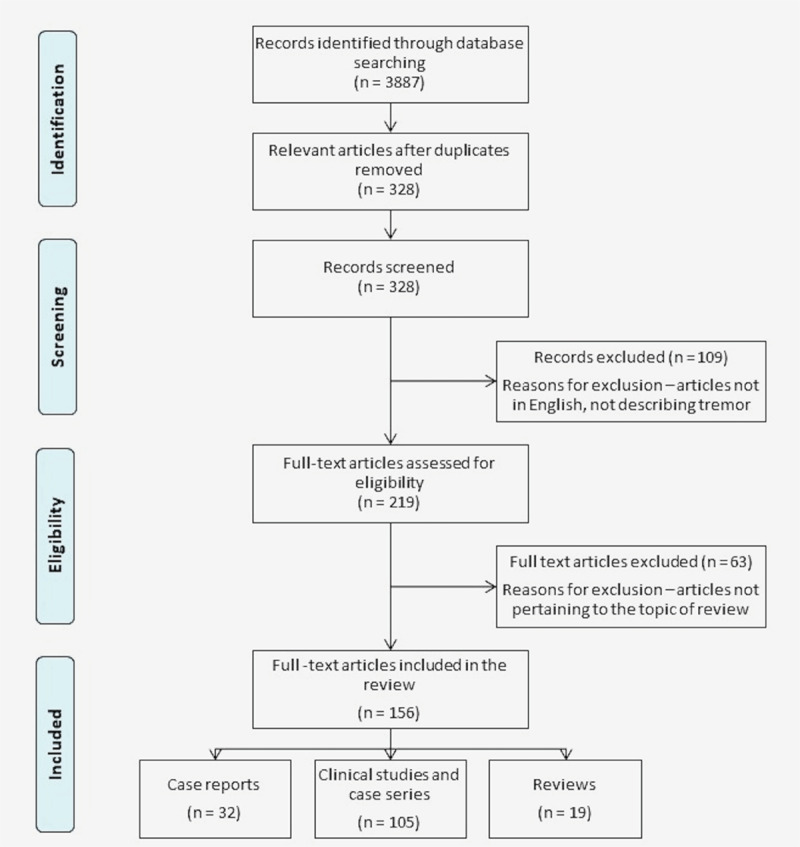
Flowchart illustrating the selection process of studies on tremor in spinocerebellar ataxia.

The authors conducted a literature search on PubMed in June 2023, using the different search terms [“tremor” and “spinocerebellar ataxia”], [“tremor” and “SCA”], [“tremor” and each spinocerebellar ataxia type (up to 50)], and [“movement disorder” and “spinocerebellar ataxia”] for this scoping review. Articles written in English were screened for inclusion in the review. Relevant references from the articles were also traced and appropriate articles were included. In case a patient was described more than once in literature, we combined the data where required, citing all related articles. We excluded articles retrieved by the search terms which had ambiguous clinical descriptions of tremor. The articles were re-assessed to avoid any duplications, and a final list was generated for inclusion in the review (Figure 1).

## Classification of SCA

SCAs consist of diseases caused by either repeat expansion mutations, or conventional nonrepeat mutations. While molecular diagnosis is the current standard for SCAs, a clinical description based on Harding’s classification of autosomal dominant cerebellar ataxia (ADCA) remains useful [5]. In the present context of nearly 50 SCAs, clinically ADCA type 1 consists of cerebellar ataxia with additional signs (SCAs 1–4, 8, 10, 12–14, 17–22, 25, 27–29, 32, 34–36, 38–40, 42–44, 46–50), ADCA type 2 comprises solely SCA7, and ADCA type 3 includes the relatively pure cerebellar ataxias (SCAs 5, 6, 11, 15(16), 23, 26, 30, 31, 37, 41, 45) [1,2]. Some of these SCAs are associated with tremor as a minor accompaniment, whereas others (such as SCA12) may present with tremor as the major manifestation. While several large series are available for the earlier SCAs, information on the newer SCAs is sometimes limited to only a few families.

### Tremor in Autosomal dominant cerebellar ataxia type 1

#### SCA1

In a study from the Clinical Research Consortium for SCAs (CRC-SCA) on 315 SCA patients (comprising SCA1, SCA2, SCA3, and SCA6), 18% had tremor, with the least prevalence in SCA1 (5.6%) [6]. One of the highest prevalences of tremor in SCA1 was seen in a Korean study where tremor (postural and terminal) was present in all six patients (100%), higher than SCA2 and SCA3 [7]. Another series noted intention tremor in 2/3 SCA1 patients [8]. Rarely, rest tremor may be present in SCA1 [9]. Even the early signs in SCA1 may consist of limb, and occasionally, lingual tremor [10]. A report from a SCA cohort documented an association between postural tremor and ataxia progression [11]. While the rate of ataxia progression was faster in SCA2 with postural tremor, a reverse trend was seen in SCA1 and SCA6, and there was no such correlation in SCA3 [11]. The presence of tremor had no association with the age of ataxia onset, gender, or age at assessment [6].

#### SCA2

The tremor was present in 49.6% of a series of 436 patients with SCA2, and it was the initial symptom in 9.7% of patients [12]. In a SCA cohort, the tremor was most common in SCA2 (31%), followed by SCA6, SCA3, and SCA1 [6]. A tremor prevalence of 30–50% in SCA2 was also noted in other studies [11,13,14,15,16,17]. However, some reports documented considerably lower [18,19,20,21,22], or higher rates of tremor [7,23]. Tremor in SCA2 was found to be associated with a longer duration of disease [12], larger CAG repeat expansion [16], and faster progression [11]. Postural or intention tremor is more common than rest tremor in SCA2 [13,14,16,17,18,19,21]. Regarding rest tremor in SCA2, some studies note scarcity [13,20,24], while others document it more consistently, often with a parkinsonian phenotype [7,9,18,25,26,27,28]. In the CRC-SCA cohort, rest tremor had a higher prevalence in SCA2 (15%) compared to the other SCAs (3–5%) [6]. In a Chinese family of SCA2 with autosomal dominant parkinsonism, most patients showed typical parkinsonian symptoms (including pill-rolling tremor in one patient), while some patients had atypical features such as cerebellar signs [25]. The patients showed good response to levodopa, except one with atypical features. Another patient, with severe 3–4 Hz rest and action tremor and parkinsonism, lacked a good response to levodopa [26].

Apart from the limbs, tremors in SCA2 may be seen involving the head, trunk, lip, tongue, and perioral regions [12,16,17,18,19]. Prominent axial tremor was found in 29% of patients with a positive correlation with CAG repeat size [29]. Head tremor was seen in 35% of SCA2 patients in one study [16], while another series noted a predominance of titubation in SCA2 compared to SCA 1 and 3 [18]. Other rare tremor syndromes in SCA2 include palatal tremor [17], and orthostatic tremor [30]. The latter was a patient with head tremor, postural proximal upper limb tremor, and on standing a high frequency (13 Hz) tremor of both lower limbs, which showed some improvement with propranolol [30].

#### SCA3

Tremor is an important component of SCA3 clinical presentation, albeit to a lesser extent than SCA2. In a study on SCA3, tremor was present in 8.3% (n = 6) of patients, and rest tremor was the commonest subtype (in 5/6 cases) [31]. Other types of tremors were postural, intention, and tremor in orthostatism (2 patients). The authors identified two distinct tremor types- a fast (6.5–8 Hz), and a slow (3–4 Hz) tremor [31]. The former was mostly a tremor in action, posture, or orthostatism, while the latter was a rest and action tremor with both distal and proximal components [31]. Other studies have documented tremor in about 10–30% of SCA3 patients [6,13,14,18,23,32]. Rest tremor and parkinsonism were noted in several studies on SCA3 [7,14,27], Genetic analysis of 60 patients with familial parkinsonism revealed SCA3 in 3 siblings (5%), who presented with levodopa-responsive rest tremor, bradykinesia, and rigidity [33]. Compared to the ataxic presentation of SCA3, the parkinsonian patients had shorter CAG repeats, and a later age of onset [33]. A clinical clue in SCA3 patients with parkinsonism is the presence of mild cerebellar oculomotor signs [13].

Postural tremor is also common in SCA3 patients [11,18,32], and a study reported action tremor in nearly three-fourths of patients [7]. The presence of postural tremor was associated with longer CAG repeat expansions in SCA3, but not in the other SCAs [11]. Ataxia progression had no association with postural tremor in SCA3 [11]. Interestingly, an association between tremor and dystonia has been described in SCA3 [6]. Lower limb tremor while sitting which disappeared on standing was reported in a SCA3 patient, and it decreased with levodopa and clonazepam [34]. Conversely, mild tremors in the lower limbs on sitting with significant aggravation on standing (tremor on orthostatism) may be an initial symptom in SCA3 [35]. This tremor (4–5 Hz) also improved with dopaminergic therapy (rotigotine patch). Another case of SCA3 showed a levodopa-responsive truncal tremor with bradykinesia and unsteady gait, and a history of truncal tremor in his father [36].

Overall, the various case series suggest that tremor is most common in SCA2, followed by SCA3 and least in SCA1 [6,13,18], And, in general, a higher prevalence of tremor in SCA was noted with worsening of ataxia, except for postural tremor in SCA1 and SCA2 [6]. However, there is considerable variation in tremor data in SCAs, and contradicting reports often deviate from this general trend.

#### SCA8, SCA10

Nearly half of the patients in the SCA8 series had tremor, with several of them having tremor as an initial symptom [37]. Interestingly, SCA8 has been associated with various parkinsonian phenotypes with rest, postural and kinetic tremors [38,39,40,41,42]. Head tremor, lingual tremor, and a combination of tremor and myoclonus have also been reported in SCA8 [42,43,44]. In SCA10, postural or intention tremor is seen in about 6% of patients [20,45].

#### SCA 12 - Special Case

SCA12, common in certain ethnic populations such as the Agarwal community in India [46], typically has tremor as a principal, often initial, manifestation, with diverse tremor phenomenology (Table 2, and Video 1) [47,48]. The first SCA12 family of German descent displayed upper limb action tremor followed by head tremor [49,50]. In a series of 49 SCA12 patients, tremor was the commonest initial symptom (73.5%), followed by ataxia (18.4%), and myoclonus (6.1%) [51]. At the time of presentation, tremor was present in nearly every patient (96%). The onset of tremor was unilateral (57%) rather than bilateral (35%), and isolated head tremor was seen in 2 (4%) patients [51]. The tremor was predominantly postural (88%), but also intentional (57%), and at rest (37%). A combination of postural and rest tremor was present in about one-third of the patients. Apart from the limbs, other types of tremors included head tremor (55%), voice tremor (43%), jaw tremor (10%) and tongue tremor (10%) [51]. A high prevalence of tremor has been noted in several other studies [48,52,53], Usually, postural and intention tremors are more common than rest tremor [43,44], and the tremor may be quite asymmetric [51,54]. Although bradykinesia and rigidity may be present in SCA12 associated with a rest tremor [51,53,55], the associated postural tremor is usually not re-emergent [54]. Head tremor is often present [49,50,51,53,56,57,58], and the other reported types include tremor of voice, jaw, tongue, orofacial, and truncal titubation [53,56,57,58,59]. In addition to tremor, dystonia is a common extrapyramidal sign in SCA12 [53], including reports of spasmodic dysphonia and cervical dystonia [56,58]. The tremor in SCA12 has been described as a dystonic tremor of the head [56], and a jerky kinetic arm tremor with dystonic posturing [58]. Interestingly, the tremor in the latter improved with alcohol and was initially labelled as ET. The clinical complexity of tremor in SCA12 is emphasized in a report showing the co-occurrence of upper limb tremor and monochorea, which is comparable to the rare phenomenology termed “bindearmchorea” [60,61].

**Table 2:** BOX: Spinocerebellar ataxia type 12: Special Case.


**BOX** **Case vignette of Spinocerebellar ataxia type 12** ***Case*** A 48-year-old male patient presented with a gradually progressive unsteadiness of gait and tremor of both upper limbs for the past 5 years. His tremor progressed to the extent that he had to take support with both hands while writing or drinking. He belonged to the Agarwal community, and his mother, maternal uncle and grandfather had similar history of tremulousness and gait unsteadiness. Video 1 shows ataxia with impaired tandem gait, and tremor in both upper limbs with postural and intentional components. ***Comment*** This patient, belonging to the Agarwal community, showed prominent tremor with ataxia, and a positive family history. This clinical presentation was suggestive of SCA12, and genetic study confirmed a CAG repeat expansion mutation in the *PPP2R2B* gene responsible for SCA12. Along with the ataxic gait, tremor was a significant and troubling symptom.

**Characteristic features of Spinocerebellar ataxia type 12** Spinocerebellar ataxia type 12 (SCA12) is common in certain ethnic populations such as the Agarwal community in India Tremor is the key clinical feature, present almost universally, and is often the initial manifestation Most common form of tremor is postural (action) tremor in the upper limbs Ataxia may not be prominent initially, hence misdiagnosed as essential tremor Tremor may start unilaterally, and remain asymmetric Rest tremor (with or without bradykinesia) is frequently present (associated postural tremor is usually not re-emergent) Other forms of tremor include head tremor, and less frequently voice, jaw, lingual, and orofacial tremors, and truncal titubation Dystonia is commonly associated, including dystonic tremor of the limbs and head Neuroimaging shows prominent cortical and cerebellar atrophy


**Video 1:** A 48-year-old male patient of spinocerebellar ataxia type 12. He has impaired tandem gait, and tremor in bilateral upper limbs with postural and intentional components.

Thus, tremor is the predominant clinical feature, seen in almost every patient, and may be the presenting symptom instead of ataxia. In this tremor-predominant scenario, the absence of prominent ataxia, as may be the case in the early stages of SCA12, can lead to an erroneous diagnosis of ET. In SCA12, tandem gait is usually impaired. However, this solitary additional feature in a patient with bilateral upper limb action tremor is also compatible with an ET plus phenotype. Thus, along with detailed phenotyping, a genetic diagnosis is essential with a high index of suspicion, especially in specific ethnic populations.

#### SCA14, SCA17, SCA18, SCA19(22), SCA20

The tremor in SCA14 is usually a postural hand tremor, or tremor of the head, trunk, or lower limbs [62,63,64,65,66,67]. The tremor is often dystonic [62,63,65], or associated with myoclonus (upper limbs, trunk, and head) [62,63]. Parkinsonism is also described, although with bradykinesia and gait freezing rather than rest tremor [67]. Clonazepam showed some efficacy in a patient with multifocal myoclonus and a 5–6 Hz dystonic truncal tremor with cervical dystonia [63].

A systematic review of genotype-phenotype correlation comprising 346 patients with SCA17 (*ATX-TBP*) did not note any pure tremor presentation [68]. In an earlier study, nearly two-thirds had parkinsonism, but the tremor was less conspicuous [69]. In 264 patients with Parkinson’s disease (PD), SCA17 was detected in one patient with rest tremor [38]. A patient of SCA17 was reported with fine postural upper limb tremor [70], while another patient manifested bilateral postural dystonic tremor in both upper limbs, which was more proximal than distal [71].

A report of a single patient of SCA18 noted an isolated palatal tremor with voice tremulousness without any ear clicking or gait ataxia [72]. Interestingly, the brain MRI of the patient showed hypertrophic olivary degeneration along with cerebellar atrophy. SCA19/ SCA22 may present with a slow frequency, irregular postural tremor of the upper limbs [73]. This was preceded by a head tremor in one patient, whose mother also had jerky neck movements [73]. Another patient presented with lower limb tremor and an unsteady gait [74]. SCA20 was identified in an Australian family, presenting with dysarthria and isolated dentate calcification on neuroimaging [75,76]. Palatal tremor (1.5–3 Hz) was evident (without ear click) in the patients, sometimes with synchronous involvement of the lips. Postural and kinetic tremor of the upper limbs, and head tremor were also observed [75,76].

#### SCA 21, SCA27, SCA29

Patients with SCA21 may present with action (postural and intention) and/ or rest tremor of the upper limbs [77,78,79,80]. The adult-onset phenotype is usually a progressive ataxia, sometimes associated with parkinsonism which is not responsive to levodopa [77]. Distal polyminimyoclonus as well as proximal myoclonus may be present [79]. Additionally, dystonic posturing in one hand was observed in a patient of SCA21 who presented with tremor in the upper limbs and head [81].

*FGF14*-related ataxia bears considerable phenotypic variability, including SCA27, episodic ataxia, and GAA repeat expansion associated late-onset cerebellar ataxia (LOCA) [82,83,84,85,86,87,88,89]. The current OMIM nomenclature for the *FGF14* mutation-associated ataxia is SCA27 A, and the GAA repeat expansion-related disorder is SCA27B [90,91]. The initial reports on SCA27 revealed childhood onset postural upper limb tremor (high-frequency small amplitude), often as the presenting symptom, aggravated by physical exercise or emotional stress [82,83]. The patients also showed head tremor, and orofacial dyskinesia. In addition to the action (postural and intention) tremor [82,83,84], a parkinsonian phenotype with rest tremor has also been described, which showed improvement with amantadine and levodopa [85]. Another series noted polyminimyoclonus in patients of SCA27 with prominent tremor [86]. Cervical dystonia was also present in one patient [86]. In *FGF14*- related episodic ataxia, upper limb postural tremor was frequently noted [87]. Postural tremor was present in 16% of patients with *FGF14* GAA repeat expansion associated LOCA [88].

SCA29 is an allelic disorder to SCA15, and usually presents as a cerebellar ataxia with infantile-onset motor developmental delay and cognitive impairment [92,93]. Action tremor (postural and intention) is commonly seen in these patients [92,93,94].

#### SCA 35, SCA36, SCA40, SCA42, SCA48, SCA50

Upper limb postural and intention tremor is commonly observed in SCA35 [95,96]. The dystonic component is sometimes noted in SCA35, either in the form of dystonic limb tremor (with voice and head tremor) [97], or cervical dystonia [96]. Postural tremor is described in about 30% of SCA36 patients [98].

A tremor-dominant phenotype was documented in SCA40, with an asymmetric upper limb postural and intention tremor, along with a side-to-side head tremor, voice tremor, and mild ataxia [99]. The mother of this patient also had a mild head tremor. Other reports on SCA40 have noted intention tremor [100,101]. Rest and action tremor with a parkinsonian phenotype is also described [102,103]. A series of SCA40 patients with familial ataxia, tremor (upper limb and head), parkinsonism, and cognitive impairment indicates a more complex presentation [103]. In SCA42, postural, rest, and head tremor (with cervical dystonia in one patient) are reported [104,105,106,107]. The rest tremor may be present without significant bradykinesia or rigidity [104,106]. A SCA42 patient manifested rest tremor (especially head tremor), ataxia, and dysarthria, along with rest tremor in her twin sister [104]. The tremor showed remarkable improvement with Zonisamide [104]. The spectrum of movement disorders in SCA48 includes chorea (62.5%), parkinsonism (62.5%), dystonia (37.5%), and tremor (37.5%) [108]. The tremor may be a postural limb tremor, head tremor, or lingual tremor [108,109]. Intriguingly, the pairing of psychiatric and cognitive symptoms with the movement disorders in SCA48 suggests a Huntington disease-like phenotype [108,109]. The newest addition to the SCA family is *NPTX1*-associated ataxia, also termed SCA50 (OMIM) [110]. It manifests postural tremor of the limbs (head tremor in one patient) and is associated with myoclonus [111].

### Tremor in autosomal dominant cerebellar ataxia type 2 (SCA7)

Intention tremor is commonly seen in SCA7 [112,113]. Postural tremor is also reported in several studies [7,23]. There was a comparable presence of postural tremor and parkinsonian symptoms (about 20%) in a series of 71 patients with SCA 7 [114]. Head tremor was noted in one patient [115]. Ocular and palatal myoclonus was reported in another patient [116].

### Tremor in autosomal dominant cerebellar ataxia type 3

#### SCA5, SCA6

In a series of 15 patients with SCA5, five had intention tremor, and two showed rest tremor which were more prominent on the action [117]. Infantile SCA5 cases may also manifest intention tremor [118,119].

Both postural and rest tremors are seen in SCA6 [6,7,9,13,23]. In the study from the CRC-SCA, 22% of the SCA6 patients had tremor, which was higher than SCA1 and SCA3 [6]. Myoclonus may accompany the action tremor in SCA6 [120]. In a series of seven SCA6 patients, three showed a nodding head tremor [120]. Intriguingly, a patient of SCA6 who underwent pancreaticoduodenectomy, developed a refractory head tremor after receiving anesthesia and metoclopramide [121].

#### SCA15(16)

In SCA15/SCA16, postural tremor of the upper limbs and trunk, and head tremor are described [122,123]. Overall, tremor was noted in 46.7% of cases, mostly postural or intention, and rarely at rest [124]. Head tremor is noted in about one-third of patients [125,126]. *ITPR1* (the gene involved in SCA15 and SCA29) missense mutations were detected in 6.66% (4/60) of patients with sporadic infantile-onset, cerebellar ataxia. All four patients had postural tremor of the arms, head, and trunk [127].

#### SCA23, SCA31, SCA37

Tremor is seen in nearly 30% of patients with SCA23 [128], characteristically as a postural tremor of the upper limbs and head tremor [129,130,131]. Rarely there may be rest tremor, and neck posturing [129,130,131]. SCA31 usually presents with a relatively pure form of cerebellar ataxia, with tremor in a few (about 4%) patients [132]. A series of five SCA31 patients with nigrostriatal dopaminergic dysfunction, noted two patients with tremor [133]. Postural tremor is present in about 30% of patients with SCA37 [134].

## Pathophysiology of Tremor in SCA

**Figure 2:**
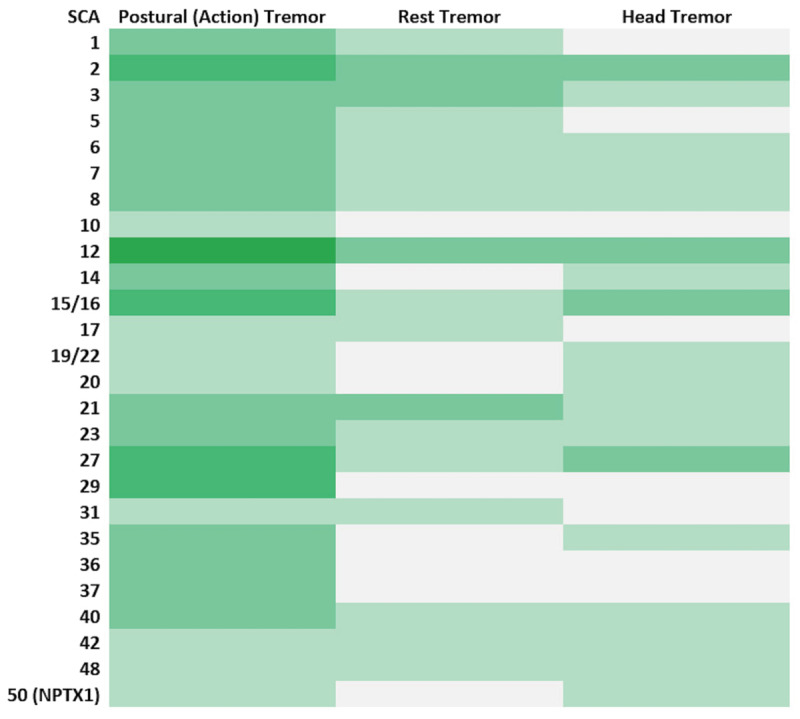
Heatmap depicting the presence of tremor in spinocerebellar ataxia.

Tremors in SCAs display a diverse phenomenology (Table 1). The prevalence of the different types of tremors varies among the SCAs, with tremor being exceedingly common in some, such as SCA12 (Figure 2). While postural or action tremor is the commonest presentation, several SCAs also manifest rest tremor, and several other tremor types.

**Table 1:** Types of tremors observed in spinocerebellar ataxia.


SCA	TREMOR				REMARKS

	POSTURAL (ACTION)	REST	HEAD	OTHER	

**1**	++	+	–	Lingual, Lip	–

**2**	+++	++	++	Truncal Lingual, Lip, Perioral Palatal Orthostatic	Levodopa responsiveness present in patients with typical parkinsonian features Rate of ataxia progression faster in SCA2 with postural tremor

**3**	++	++	+	Truncal Tremor on orthostatism	Levodopa responsiveness present in the ‘slow’ tremor Association present between tremor and dystonia

**5**	++ (Predominantly Intention)	+	–	–	–

**6**	++	+	+	–	Myoclonus may accompany action tremor

**7**	++ (Intention and Postural)	+	+	Ocular and palatal myoclonus (tremor)	–

**8**	++	+	+	Lingual	Combination of tremor and myoclonus Presentation with parkinsonian phenotypes– PD, PSP, CBS, MSA–C

**10**	+ (Intention and Postural)	–	–	–	–

**12**	++++	++	++	Voice Lingual, jaw Orofacial Truncal	Tremor may be the initial symptom Tremor is often asymmetric Dystonia may be present– dystonic tremor, spasmodic dysphonia, cervical dystonia

**14**	++	–	+	Truncal	Dystonic tremor, cervical dystonia Myoclonus– upper limbs, trunk, and head

**15/16**	+++	+	++	Truncal	–

**17**	+	+	–	–	Tremor associated with dystonia or parkinsonism

**18**	–	–	–	Palatal	Single case report of isolated palatal tremor

**19/22**	+	–	+	–	–

**20**	+	–	+	Palatal Lip	Palatal tremor is frequently present

**21**	++	++	+	–	–

**23**	++	+	+	–	–

**27**	+++	+	++	–	Tremor may be the initial symptom Postural tremor frequently noted in *FGF14*– related episodic ataxia Postural tremor present in 16% of patients with *FGF14* GAA–LOCA

**29**	+++ (Intention and Postural)	–	–	–	Although allelic disorder to SCA15, head tremor is not commonly reported

**31**	+	+	–	–	–

**35**	++ (Intention and Postural)	–	+	Voice	Dystonic limb tremor, cervical dystonia

**36**	++	–	–	–	–

**37**	++	–	–	–	–

**40**	++ (Intention and Postural)	+	+	Voice	–

**42**	+	+	+	–	Cervical dystonia with dystonic head tremor is reported

**48**	+	+	+	Lingual	–

**50 (NPTX1)**	+	–	+	–	Myoclonus associated with tremor


+ Rare (or case reports), ++ Common, +++ Very common, ++++ Nearly always. SCA- Spinocerebellar ataxia.

**Figure 3:**
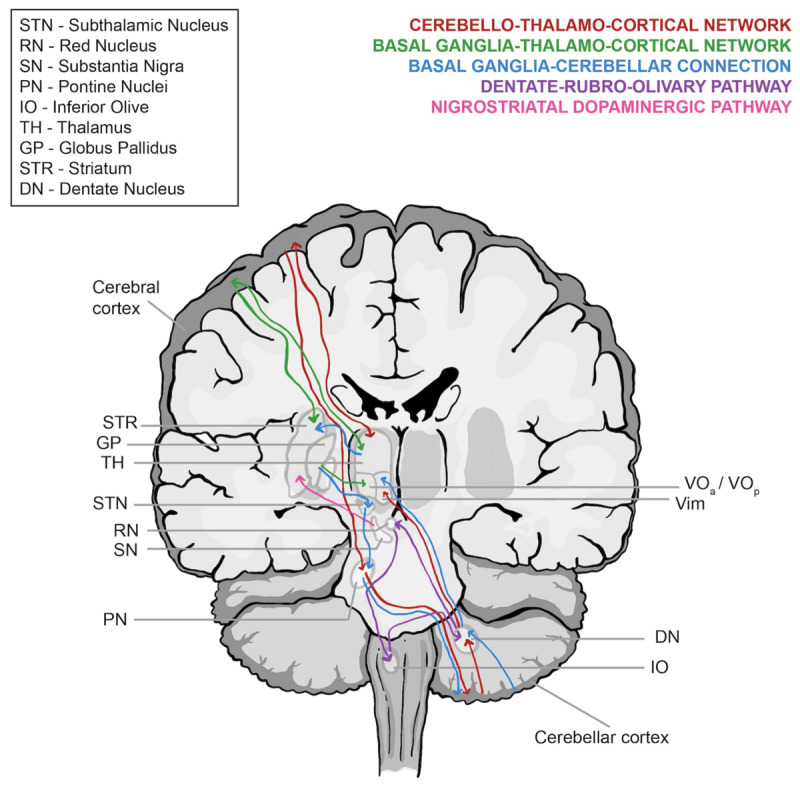
Pathophysiology of tremor in spinocerebellar ataxia (SCA). The cerebellum, mainly via the cerebello-thalamo-cortical circuit, plays a key role. Nigrostriatal dopaminergic dysfunction is associated with parkinsonism and rest tremor in SCA. A dystonic tremor in SCA may additionally involve the basal ganglia-thalamo-cortical pathway. Other possible pathways associated with tremor in SCA include the bidirectional cerebello-basal ganglia connection, and the dentate-rubro-olivary circuit.

Tremors in SCAs bear important pathophysiological connotations linked to the cerebellar networks, and it helps to substantiate the role of the cerebellum as a primary driving force for tremor. While the cerebello-thalamo-cortical (CTC) circuit is central to the tremor in SCAs, additional pathways comprising the basal ganglia and the striatonigral dopaminergic system are also probably involved (Figure 3). Postural tremor is frequently present in SCAs 2, 3, 12, 15(16), 27, 29, and several others. Cerebellar pathology is a common feature in the SCAs [135,136], and its association with action tremor further substantiates the role of the cerebellar connections, especially the CTC circuit, in such tremor. In SCA12, which is phenotypically a close mimicker of ET, neuroimaging shows prominent cortical and cerebellar atrophy, and functional neuroimaging has revealed a pattern of cortical and cerebellar hypometabolism [55]. Modulation of the CTC circuit by deep brain stimulation (DBS) of the ventral intermediate nucleus (Vim) of the thalamus has been used successfully for treating tremor in SCAs such as SCA2, SCA6, and SCA31 [26,27,71,137], Moreover, magnetic resonance-guided focused ultrasound (MRgFUS) targeting the CTC tract ameliorated a refractory upper limb action tremor in a patient with SCA12 [138]. Vim is related to the cerebellar connections with the motor cortex, and the positive effect of Vim DBS and CTC MRgFUS in SCA corroborates with the proposed tremor network. Another network associated with tremor is the dentate-rubro-olivary circuit related to the inferior olivary nucleus (ION). Involvement of the inferior olive has been documented in SCAs such as SCAs 1, 2, and 3 [135]. Hence, this network may be involved in tremor-related activity in SCA as well. However, a lack of direct association studies with tremor in SCA keeps this notion hypothetical. Interestingly, a pilot trial on dentate nucleus DBS which included SCA3 patients, observed improvement in tremor but not in ataxia [139]. These possible mechanisms for tremor in SCAs highlight the role of the cerebellum.

Parkinsonism with rest tremor is seen in SCAs such as SCA2 and SCA3, and neuroimaging has revealed striatal dopaminergic dysfunction in both diseases [140]. The rest tremor in such cases is usually responsive to levodopa. Thus, it indicates dysfunction of both the cerebellum and the basal ganglia and is reminiscent of the dimmer-switch model of rest tremor [141]. However, reports on dopaminergic dysfunction in SCA12 are contradictory, with one study revealing normal dopaminergic function in the presence of rest tremor [19,55,142], Hence, this suggests the possibility of the rest tremor having a different mechanism. Dystonia is commonly reported in SCA12, and the rest tremor may have a dystonic component. The cerebellar networks appear to be the primary pathophysiological substrate for the various tremor manifestations in SCA12, including rest tremor.

Dystonia and dystonic tremor are encountered in SCAs such as SCAs 3, 12, 14, 17, 21, 35, and 42. In addition to the cerebellum, basal ganglia involvement is evident in several SCAs [135]. Interestingly, Vim DBS did not produce persistent improvement in dystonic hand tremor in a patient of SCA35 [97]. Rather, the dystonic limb tremor in a patient of SCA17 improved markedly with DBS of bilateral globus pallidus internus (GPi) [71]. Pallidal stimulation was partially effective on the dystonic tremor in a SCA14 patient [65]. This indicates the involvement of both the cerebellum and the basal ganglia circuits. This is congruent with the network-level hypothesis suggested in dystonic tremor which implicates a wide network comprising both the CTC and the basal ganglia-thalamo-cortical pathways [141,143]. Current research has emphasized the role of the cerebellum in dystonia and dystonic tremor [144,145], and recently, a direct bidirectional connection between the cerebellum and the basal ganglia has also been proposed [146]. In this context, SCA12 presents an intriguing situation where, although dystonic tremor is often present, atrophy of the caudate and putamen is yet to be established [48,147]. This further strengthens the role of the cerebellum and its connections in dystonic tremor.

Thus, the SCAs present a unique opportunity to study the effect of cerebellar dysfunction on tremor. Despite the possible involvement of other structures such as the basal ganglia, the cerebellum appears to be the key substrate for tremor in SCA- postural, rest, or dystonic. Large-scale studies on detailed phenomenology and underlying pathophysiology are required for the definitive characterization of tremor in SCAs.

## Management of Tremor in SCA

Although the list of SCAs has witnessed a continuous numerical expansion, it has not yet been translated into a definitive therapeutic modality. Regarding tremor in SCAs, there is a scarcity of clinical trials addressing this specific issue, and hence, information has to be derived from clinical studies, case series and reports.

### Medical management

Various medications have been used to treat tremor in SCAs, with inconsistent results. In general, treatment is usually driven by the phenomenology of tremor and associated clinical features. A postural tremor, with its resemblance to that of ET, has often been treated likewise. The postural tremor in SCA12 showed improvement, at least in some patients, with beta-blockers (propranolol), primidone, and possibly clonazepam [148,149]. A study on SCA12 noted a subjective improvement of tremor and ataxic symptoms, using varying combinations of medications depending on the phenomenology (tremor, spasticity, rigidity, and dystonia) [51]. Amantadine and propranolol were most frequently used, followed by clonazepam, primidone, levodopa, trihexyphenidyl, and baclofen. However, the postural tremor may be refractory to medical management [59,99]. Propranolol was effective in treating orthostatic tremor in a patient of SCA2 [30]. Clonazepam proved useful in a patient of SCA14 with a dystonic truncal tremor associated with myoclonic jerks [63].

Rest tremor in SCAs, especially when associated with parkinsonism, is usually treated with levodopa. The response is sometimes robust enough to prompt an initial diagnosis of PD, as seen in SCA2 and SCA3 [25,33,150,151,152]. Similar improvement is also seen in other SCAs [38,85]. However, it is not universal, and a good response to levodopa may remain elusive [26,40,77], Levodopa was better at reducing a ‘slow’ (3–4 Hz) tremor in SCA3 compared to a ‘fast’ (6.5–8 Hz) tremor [31]. Levodopa was also effective in lower limb tremor and truncal tremor in SCA3 [34,36]. Tremor on orthostatism in a SCA3 patient showed improvement with clonazepam, trihexyphenidyl, and markedly with rotigotine patch [35]. Dysfunction of the nigrostriatal dopaminergic system has been demonstrated in SCAs using functional neuroimaging [31,33,35,36,153,154]. This might explain the beneficial role of levodopa.

A patient of SCA42 (*CACNA1G*) manifested prominent rest tremor (including head tremor) which improved with zonisamide at a low dose (25 mg/ day) [104]. *CACNA1G* encodes the voltage-dependent T-type calcium channel (TTCC) Ca_v_3.1 [155,156]. Zonisamide is a TTCC blocker (and a sodium channel blocker) and was found to ameliorate the voltage dependence of Ca_v_3 [1]. mutation seen in SCA42 [155]. TTCCs are abundant in structures related to the cerebello-thalamo-cortical and basal ganglia-thalamo-cortical circuits, and perturbations in the TTCC activity are linked with ET and PD [156]. Trials of TTCC blockers such as Suvecaltamide are being conducted in ET. A positive outcome would unfold a novel avenue of treatment for tremor, and with the good effect of zonisamide in SCA42, future studies with TTCC blockers might be contemplated in SCAs as well.

### Botulinum neurotoxin injection

Cervical dystonia with head tremor and a spasmodic adductor dysphonia in SCA12 improved with Botulinum neurotoxin (BoNT) injection [58]. The father of the proband also had head tremor which responded to BoNT injections. A similar result was reported in SCA8. The palatal tremor in a SCA18 patient was ameliorated by BoNT injection in the tensor veli palatini muscle without significant adverse effect [72]. This indicates that BoNT injections may be useful in head tremor, and possibly other forms of dystonic tremor seen in SCA.

### Surgical management

#### Deep Brain Stimulation Surgery

In SCAs, DBS may be useful for tremor refractory to medications [26,27,71,137], The Vim nucleus of the thalamus is the commonest target for tremor in SCAs [26,27,71,137], Vim DBS was effective in a SCA2 patient with rest and action tremor, and another with a coarse action tremor [26,27]. DBS with a subthalamic-thalamic electrode position ameliorated a debilitating postural tremor in SCA2 [157]. Thalamic surgery also resulted in improvement of tremor in SCA6 and SCA31 patients with intractable action tremor [137]. GPi DBS significantly reduced a dystonic limb tremor in SCA17 [71], and was partially effective on dystonic tremor in SCA14 [65]. A SCA3 patient with levodopa-responsive parkinsonism including rest tremor, showed remarkable improvement with bilateral subthalamic nucleus (STN) DBS [158]. Recently, dentate nucleus DBS for cerebellar ataxia was evaluated in a randomized pilot trial on five patients (which included SCA3), and improvement of tremor was noted, despite lacking a significant effect on ataxia [139].

Reports on thalamic DBS in ET had raised the concern of worsening ataxia. A study on 113 ET patients who underwent Vim DBS, documented stimulation-related ataxia in 35% of cases [159]. A ventrocaudal stimulation in the sub-thalamic area was found responsible for the progressive gait ataxia in ET patients [160]. This seems a little disconcerting for Vim DBS in SCAs. A patient of SCA2 undergoing unilateral Vim DBS developed a transient post-operative worsening of ataxia, which recovered to the pre-operative baseline with rehabilitation [71]. In a series on Vim DBS in SCAs, neither improvement nor worsening of ataxia was noted [137]. Thus, Vim DBS does not appear to have a persistent benefit on ataxia in SCA. However, further research is required to evaluate the outcome of DBS in SCAs, along with the delineation of the ideal target and stimulation parameters.

#### Magnetic resonance-guided focused ultrasound

MRgFUS thalamotomy is an emerging option in the treatment of tremor in ET and PD [161,162]. A recent report utilized unilateral MRgFUS targeting the cerebello-thalamo-cortical tract to successfully treat a refractory upper limb action tremor in a patient of SCA12 [138]. The patient noted post-operative gait impairment, which resolved within three months [138].

### An approach to the management of tremor in SCA

In the absence of any large-scale study specific to the treatment of tremor in SCAs, its management is mostly guided by phenomenology. A prominent postural tremor may be initially treated with beta-blockers (propranolol). Although primidone is also used, its effect on the worsening of ataxia may limit its use. Clonazepam, trihexyphenidyl, and baclofen may be useful, especially in dystonic tremors. Clonazepam is also effective in the presence of myoclonus. Levodopa is the preferred initial medication for rest tremor in SCAs. Levodopa may be effective in dystonic tremors as well. Zonisamide is useful in controlling tremors specifically in SCA42. BoNT injections may be utilized for head tremor, and dystonic tremors. For treatment-refractory tremors, surgical management in the form of DBS, or MRgFUS may be useful. However, its effect on any worsening of the ataxia requires further research.

## Conclusion

Tremor is an elemental component of SCAs, and in some of them, such as SCA12, tremor may be the initial presenting symptom. While action tremor is the most common type, rest tremor and other rare tremor syndromes have also been reported. The multifarious phenomenology and underlying pathophysiology of tremor in SCAs is indeed intriguing. The cerebellum appears to be the primary substrate for the different types of tremors via its various networks such as the CTC circuit, and connections with the basal ganglia. However, further studies are required for a detailed description of the clinical manifestations, elucidation of the pathophysiology, and effective therapeutic modalities regarding the distressing symptom of tremor in SCAs.
